# A short assessment of health literacy (SAHL) in the Netherlands

**DOI:** 10.1186/1471-2458-14-990

**Published:** 2014-09-23

**Authors:** Henk Pander Maat, Marie-Louise Essink-Bot, Karlijn EF Leenaars, Mirjam P Fransen

**Affiliations:** Department of Languages, Literature and Communication, Utrecht Institute of Linguistics OTS, Trans 10, 3512, JK Utrecht, The Netherlands; Department of Public Health, Academic Medical Centre, University of Amsterdam, Meibergdreef 9, 1105, AZ Amsterdam, The Netherlands; WU Social Sciences, Health and Society, Hollandseweg 1, 6706 Wageningen, The Netherlands

**Keywords:** Health literacy, Testing, Word recognition, Reading comprehension

## Abstract

**Background:**

An earlier attempt to adapt the REALM (Rapid Estimate of Adult Literacy in Medicine) word recognition test to Dutch was not entirely successful due to ceiling effects. In contrast to REALM, the Short Assessment of Health Literacy (SAHL) assesses both word recognition and comprehension in the health domain. The aim of this study was to design, test and validate a SAHL for Dutch patients (SAHL-D).

**Methods:**

We pretested 95 health-related terms (n = 127) and selected 33 best performing items for validation in a quantitative survey (n = 329). For each item, a correct recognition (1 point) and comprehension (1 point) contributed to the total score (scale 0–66). Internal consistency was assessed using Cronbach’s alpha. Construct validity was examined by analyzing association patterns of SAHL-D with educational level, objective and subjective health literacy, prose literacy, and vocabulary. Receiver operating characteristic (ROC) curves, with prose literacy as the reference standard, determined optimal cut-off scores.

**Results:**

Cronbach’s alpha was 0.77 for recognition, 0.79 for comprehension, and 0.86 for the total score. Scores significantly differed substantially by educational level. Association patterns mostly confirmed a priori expectations in direction and strength, thereby supporting the construct validity of the SAHL-D. The optimal cut-off scores for differentiating between adequate and low literacy lie between 52.5 and 55.5. A shorter SAHL-D version presenting 22 terms offers a comparable prediction performance.

**Conclusion:**

The results provide positive evidence for the reliability and validity of the SAHL-D. The SAHL-D can be applied to analyze the role of health literacy in health and healthcare, and for the development and evaluation of targeted interventions.

**Electronic supplementary material:**

The online version of this article (doi:10.1186/1471-2458-14-990) contains supplementary material, which is available to authorized users.

## Introduction

In our current information society, individuals are increasingly required to participate in complex decision-making processes. For example, managing health and finances involves obtaining and processing complex information, and making decisions in interaction with domain experts such as physicians and financial planners. To succeed in these tasks, individuals need to be ‘literate’ in various ways. Rapid and reliable assessments of these literacy levels are needed, not only to help professional communicators, but also to study the effects of literacy deficiencies and evaluate literacy-focused interventions. This paper presents a new health literacy assessment for Dutch patients.

## Background

In its general sense, literacy refers to the ability to read and write. At the basic level, this ability is associated with reading fluency and word recognition as measured by standard reading tests. At an advanced level, this ability is associated with vocabulary, i.e. knowledge of word meanings. Both word recognition and vocabulary are essential for reading comprehension [1]. A broader notion is adult functional literacy [2], which covers three subskills required in everyday life, independent of topic domains: prose reading, comprehending diagrams, and doing computations. The central skill when it comes to using health information seems to be prose reading, i.e. making sense of texts. This requires not only lexical knowledge, but higher-order processes such as contextual meaning construction as well.

In addition to these general literacy concepts, there is a growing interest in domain-specific literacies, which has provided concepts such as financial literacy [3], media literacy [4] and health literacy (HL) [5]. The definitions of these concepts vary considerably.

In the field of HL, broad conceptual definitions go hand in hand with specific operational definitions [6, 7]. In a content analysis of the HL literature, Sørensen et al. [8] distinguished between accessing, understanding, appraising and applying health-related information. Nutbeam [9] proposed the following levels of HL: 1) basic reading and writing skills needed to understand health information (functional HL); 2) advanced cognitive, social and literacy skills needed to communicate about health (interactive HL); and 3) advanced cognitive, social and literacy skills needed to critically analyze and apply health information in one’s own situation (critical HL).

Valid and reliable measurement of HL is essential to investigate the impact of low HL on population health and healthcare use, to analyze the differential effectiveness of health interventions by HL level, and to develop, evaluate and implement effective evidence-based interventions targeting people with low HL. Clinical applications of HL assessment intend to enable clinicians to effectively adapt their communication strategies to patients with low HL. Brief and easy-to-use HL measures have been developed in English, including the Rapid Estimate of Adult Literacy in Medicine (REALM) [10]. Fransen et al. [11] adapted the REALM by translating the 66 English words into Dutch (REALM-D) [11]. Although the REALM-D proved to be feasible and reliable, it did not differentiate between intermediate and higher education levels. Of these latter groups, the proportions correct were high (94% and 97%, respectively) and even the low-educated group scored 87%, suggesting that the test suffers from a ceiling effect. Interestingly, Nurss et al. [12] and Lee et al. [13] had similar experiences in constructing a Spanish version of REALM: highly skewed distributions with a large majority of the scores being ≥ 90% [12, 13]. Nurss et al. [12] explained this by pointing out that Spanish has a more regular correspondence between graphemes and phonemes (letters and sounds) than English, so that Spanish words are relatively easy to pronounce. To overcome this problem, Lee et al. [13] introduced a semantic component in their word-based test. First, they developed the SAHLSA (Short Assessment of HL for Spanish-speaking Adults), which was later supplemented by an English version (SAHL-E) [13, 14]. For every term, the participant has to choose between two words, of which only one is meaningfully related to the term. To use an example from the later English version SAHL-E, *kidney* had to be associated with either *urine* or *fever*. In order to receive one point for an item, both the pronunciation and the association had to be correct. The SAHLSA produced a more balanced score distribution, was reliable and unidimensional, and correlated well (Pearson 0.65) with the Test of Functional Health Literacy in Adults (TOFHFLA). Lee et al. also presented an 18-item version of the SAHLSA [14].

Since Dutch resembles Spanish in its relatively transparent orthography, adding a semantic component to a pronunciation task is assumed to produce a more powerful Dutch HL measure than the REALM-D. The aim of this study was to design and test a SAHL for Dutch patients (SAHL-D), as well as to validate it against various other literacy measures, including a prose comprehension test.

## Methods

### Pretest

The authors HPM and MF selected 95 candidate SAHL-D terms from a Dutch thesaurus of health terms http://www.thesauruszorgenwelzijn.nl [15], of which 20 were related to medical specialties, tests and treatments (e.g. oncology, defibrillation), 15 to bodily functions and health behaviors (e.g. biorhythm, hygiene), 25 to the human body (e.g. pigment, pancreas) and 35 to diseases and symptoms (e.g. embolus, hemophilia). The chosen terms were potentially relevant to a general public. We avoided acronyms and terms referring to phenomena only known to medical professionals and particular patient groups. All terms were provided with a correct and an incorrect association word, using medical dictionaries when necessary. For example, ‘hemophilia’ could be associated with ‘clotting’ (correct) or ‘immunity’ (incorrect). The target word, the two associates and a ‘Do not know’ option were presented on cards, using large print.

Potential participants for the pretest were approached by undergraduate students (Language and communication) in the waiting room of the outpatient clinic of Internal Medicine at a large university hospital. Inclusion criteria were aged ≥ 18 years and able to communicate in Dutch. Those willing to participate signed an informed consent form, filled in a questionnaire and participated in a personal interview with one of the students.

The questionnaire assessed general vocabulary skills based on a written multiple choice vocabulary test used in the 8th grade of Dutch pre-vocational secondary education [16]. Each item presents a sentence with one word underlined; the respondent has to choose the correct meaning of that word from the four possible meanings that are offered.

In the personal interview, the SAHL-D was administered by handing the participant the 95 cards, one by one. Word recognition was assessed by asking the participant to read the word out loud. The instructions for students contained information on correct phonetic pronunciation and the correct stress of each syllable in each word. Word comprehension was assessed by asking participants to choose the correct word associated with the ‘target’ word, or to use the ‘Do not know’ option; participants were encouraged not to guess the answer.

In the pretest we analyzed item scores and distributions of proportions correct to select the items with the best discriminative ability. Reliability of the set of 95 items was analyzed by Cronbach’s alpha. Analyses of variance (ANOVA) were used to assess relations between educational level and scores. The feasibility was assessed by noting the administration time for a subset of participants. Finally, we examined whether word features (such as opaque orthography and corpus frequency) were related to recognition and comprehension of each word.

### Main study

We selected a subset of the pretest item pool by rejecting items that were scored correctly for recognition or comprehension by at least 95% of the participants. This left 33 items that mainly refer to medical specialties, tests and treatments on the one hand, and diseases and symptoms on the other (Additional file 1). Most of the terms referring to body parts, bodily functions and health behaviors did not meet the inclusion criteria. We then constructed a more demanding semantic test component. To assess word comprehension, instead of presenting 2 associated words we decided to present 3 candidate meanings of each word (1 correct, 2 distractors), together with a ‘Do not know’ option. As illustrated in Additional file 2, each item presents a distractor that is more or less related and a distractor that more obviously incorrect. Whereas the semantic test component in the pretest measured ‘surface-level familiarity’ (knowing which notions are related to the term and which are not), the SAHL-D aims to tap into ‘concept-level familiarity’ (knowing what the term actually refers to) [17].

Participants for the validation study were drawn from a test panel of The Netherlands Institute for Health Services Research, which is a list of people who are periodically invited to participate in various health-related research studies [18]. Inclusion criteria were age 18–75 years, and ability to read, write and converse in Dutch. Participants were approached by mail with an online questionnaire; participants were asked to indicate whether they were willing to participate in a telephone interview later on. Only data of consenting participants were used.

The following variables were assessed in the online questionnaire:

- *Background characteristics:* Gender; age; educational attainment level; ethnic background; native language; whether they work(ed) in health care; and how often they had contact with a professional care provider in the past year. Following the International Standard Classification of Education (ISCED), educational level was categorized as low (level 0–2: early childhood; primary education; lower secondary education); intermediate (level 3–5: upper secondary; post secondary; short cycle tertiary); and high (level 6–8: bachelor; master; doctoral [19].
- *General vocabulary:* In the absence of a brief vocabulary test for Dutch adults*,* we created a general vocabulary measure by selecting 50 terms typical of formal Dutch prose style, such as ‘interruption’ and ‘precarious’ , and presenting 4 alternatives together with a ‘Do not know’ option for each item; participants were encouraged to choose this latter option in case of serious doubt. In the final scale we left out 2 of the 50 items with negative rest-item correlations (due to problems with the alternatives). For the resulting 48-item test, alpha was 0.87.
- *Prose literacy:* In this study, we sought to validate our literacy measure by comparing it to a general test of higher-order reading skills, especially the contextual reconstruction of meaning in prose contexts (as opposed to word knowledge). Prose literacy was assessed by a subset of items from a reading comprehension test widely used for 9^th^ graders in Dutch pre-university secondary education (total 16 items) [16]. The test does not require specific topic knowledge. Specifically, we used four reading passages and 16 multiple choice text comprehension items about argumentative relations, relations between sentences and paragraphs, and main ideas for texts or paragraphs. Two questions ask for sentence-level paraphrases. After dropping an item with a low rest-item correlation, Cronbach’s alpha was 0.75 for the remaining 15 items. We defined adequate and inadequate prose literacy with reference to the mean proportion for the lowest educational group (0.44). We stipulated that scores ≤ 6 (corresponding to a proportion of .4) reflect inadequate prose literacy and that scores of ≥ 7 reflect adequate prose literacy.
- *Health Literacy Survey-Europe Q16*: A short version of the Health Literacy Survey-Europe [20] was used to assess subjective health literacy. The HLS-EU was derived from a theoretical model that integrates health care, disease prevention and health promotion, and four information processing stages (access, understand, appraise and apply) related to health- relevant decision-making and tasks [8].

The HLS-EU-Q16 consists of 16 items scored on a 4-point scale (very difficult to very easy). For each item the option ‘Do not know’ was also provided [20].

In a telephonic interview, NVS-D and SAHL-D were administered. These tests were sent as pdf files by email, not beforehand but upon starting the interview. As soon as the mail arrived, the participant started working on the NVS-D, followed by SAHL-D.

- *Newest Vital Sign (NVS):* The NVS is a 6-question tool to assess an individual’s ability to find and interpret information (both text and numerical information) on an ice cream nutrition label [21]. Earlier, Fransen et al. [11] translated and tested the NVS in Dutch (NVS-D); the cross-cultural adaptation and validation of the NVS-D is submitted for publication.

During the interview, we sent one file with the ice cream label and another one with the questions; respondents were asked to open both files on their screen. The interviewer read the questions out loud while the respondents read the questions and looked at the label on their screen.

- *SAHL-D*: SAHL-D started with a title page and provided a single word per page, with the candidate meanings underneath it. The participant proceeded page by page. The item order was kept on, except in rare cases when words were skipped accidentally (by pressing the arrow button more than once). In those cases, the interviewer steered the participant back to the omitted word after the current item has been completed. At any time of the test, the participant saw only a single target word on the screen. Upon opening a new page, participants were given 5 seconds to pronounce the word, after which a multiple choice option was to be chosen immediately. This procedure practically rules out the possibility of using dictionaries. The participants worked alone (possible consultations with others would have been overheard). Administration of the SAHL-D took (on average) 6.39 min*.*

In the validation study we assessed the proportions of correct answers and score distributions of the SAHL-D. Feasibility was assessed by calculating percentage refusals and acceptance and the time to complete the SAHL-D. Reliability was tested with Cronbach’s alpha.

To explore the possibility of a shorter SAHL-D, we created an item subset by first discarding recognition items with rest-item correlations of ≤ 0.10 in the 33-item reliability analysis and/or a proportion correct of ≥ 0.95. This left 22 recognition items. We included the shorter 22-item set (SAHL-D22) in the analyses to illustrate the potential for a briefer SAHL-D.

Construct validity was examined by analyzing association patterns of the SAHL-D, NVS-D, HLS-EU-Q16, educational level, prose literacy and vocabulary scores in relation to predefined expectations about the size and pattern of the associations.

The following hypotheses were formulated:

- Regarding known-groups validity, we expected the SAHL-D to be able to distinguish between low, intermediate and high levels of education based on significant differences in the mean scores.
- Because of partly overlapping constructs, we expected a strong correlation between general vocabulary, prose literacy, NVS-D and the SAHL-D.
- We expected a significant (but not sizeable) correlation between the SAHL-D (objective measure) and the HLS-EU-Q16 (subjective measure).
- Regarding associations with socio-demographic variables, earlier literacy research [22, 23] led us to expect a strong positive association between the SAHL-D and educational level, and a moderate negative correlation between SAHL-D and age; no significant gender difference was expected.

ANOVA pairwise comparisons with Bonferroni correction were used for multiple testing to test differences in the SAHL-D scores by educational level, age, gender, and profession (working in health care). The association between the SAHL-D with general vocabulary, prose literacy, NVS-D, and HLS-EU-Q16 was tested with Pearson’s correlations and stepwise linear regression analyses to correct for background variables.

We used receiver operating characteristic (ROC) curves with adequate prose literacy as the reference standard to determine optimal cut-off scores for identifying objective HL.

## Results

### Pretest

Of the 127 patients participating in the pretest, 51% was male, 20% had a low and 34% had an intermediate educational level; the age range was 20–85 years with a mean of 50.4 (SD 14.4) years.

On average, the 95-word test took 9 min. The **recognition task** proved to be relatively easy, with a mean proportion correct of 0.93. Of the 95 words, 5 were correctly pronounced by all participants and another 53 items were correct for ≥ 95% of the participants. Cronbach’s alpha for the recognition test was 0.94. The **comprehension test** was of similar difficulty (mean proportion correct 0.90). Of the 95 items, 4 were correctly scored by all participants and another 40 items were correct for ≥ 95% of the participants. Cronbach’s alpha for the comprehension test was 0.93.

The correlation between recognition performance and comprehension performance was 0.83 (Pearson *r*). Correlations between SAHL-D recognition and comprehension with general vocabulary were similar, i.e. 0.66 and 0.57, respectively. The total correct score for the candidate items varied with educational level, although the effect size was modest (F [2,122] = 4.49, p < 0.05; eta^2^ = 0.069).

### Main study

We aimed to include 300 participants in the validation study. In total 2000 individuals were invited to participate in an online survey and telephone interview; of these, 1037 filled in the questionnaire of which 595 agreed to be contacted by telephone and of which 329 finally participated in the personal interview. No significant difference in educational level was found between participants and non-participants. Mean age of participants was 56.2 years compared with 49.3 years for non-participants (p < 0.05). There was a significant difference in gender between participants and non-participants: 41% of the participants was male compared with 50% of the non-participants (p < 0.01).

Table 1 presents the characteristics of the participants in the validation study, as well as the proportions correct for recognition and comprehension. The grand means for proportions correct were 0.89 for recognition and 0.80 for comprehension (compared with 0.93 and 0.90, respectively, for the candidate item set in the pretest). Women had higher comprehension and total SAHL-D scores than men. Significant differences were found in the scores for age, education level and profession in health care. The effect of educational level on the total scores (F[2,320] = 13.82, p < 0.001; eta^2^ = 0.183) was more robust than for the pretest item set.

**Table 1:** **Background characteristics and mean SAHL scores (n = 329)**

	n (%)	SAHL recognition (SD)	SAHL comprehension (SD)	SAHL total (SD)
**Gender**				
Men	136 (41)	29.0 (3.7)	**25.2 (4.8)**	**54.2 (7.7)**
Women	193 (59)	29.4 (3.0)	**27.2 (3.7)**	**56.7 (5.9)**
**Age** mean age: 56.2 (14.9)	-			
<44	86 (26)	**29.8 (3.0)**	**26.4 (4.6)**	**56.2 (6.9)**
45-65	131 (40)	**29.6 (3.2)**	**27.1 (4.1)**	**57.7 (6.5)**
>65	112 (34)	**28.3 (3.5)**	**25.5 (4.1)**	**53.8 (6.8)**
Education*				
Low	92 (28)	**27.5 (3.7)**	**24.6 (4.4)**	**52.1 (7.0)**
Intermediate	123 (38)	**29.0 (3.3)**	**25.9 (4.3)**	**54.9 (6.9)**
High	110 (34)	**31.0 (1.8)**	**28.4 (3.2)**	**59.3 (4.2)**
**Ethnic origin**				
Dutch	313 (95)	29.3 (3.2)	26.3 (4.3)	55.6 (6.8)
Other	16 (5)	28.5 (4.2)	27.3 (3.9)	55.8 (7.5)
**Mother tongue**				
Dutch	319 (97)	29.3 (3.2)	26.4 (4.3)	55.6 (6.8)
Other	10 (3)	28.8 (4.0)	25.8 (4.1)	54.6 (7.2)
Contact with professional care provider in past year**				
0 times	28 (9)	29.2 (3.6)	26.8 (3.7)	55.9 (6.7)
1 – 5 times	196 (60)	29.2 (3.2)	26.1 (4.3)	55.3 (6.6)
5 – 10 times	55 (17)	29.4 (3.6)	26.8 (4.5)	56.2 (7.5)
> 10 times	47 (14)	29.3 (3.0)	27.1 (4.3)	56.5 (6.8)
Working in health care***				
Never worked in health care	213 (65)	**28.9 (3.4)**	**25.5 (4.2)**	**54.4 (6.7)**
Used to work in health care	56 (17)	**29.1 (3.6)**	**27.4 (4.6)**	**56.6 (7.7)**
Now works in health care	54 (16)	**30.6 (2.1)**	**28.9 (2.9)**	**59.5 (4.4)**

*4 missing.

**3 missing.

***6 missing.

[Significant differences (p < 0.05) are presented in bold].

Cronbach’s alpha’s for SAHL-D recognition, comprehension and total were 0.77, 0.79 and 0.86, respectively; for SAHL-D22, these alpha’s were .74, .73 and .83 respectively. Table 2 shows the correlations between SAHL-D22, SAHL-D33, general vocabulary, prose literacy, NVS-D, and HLS-EU-Q16. SAHL-D and SAHL-D22 showed substantial correlations with prose literacy, vocabulary and NVS-D. The total SAHL-D and SAHL-D22 scores show higher correlations with the other literacy measures than the recognition scores or comprehension scores by themselves do. Hence combining recognition and comprehension components adds precision to literacy measurement. Another indication that recognition and comprehension provide different information lies in their correlation (.63), which is substantial but far from perfect. The lowest correlations in Table 2 were those involving the HLS-EU-Q16.

**Table 2:** **Correlations between SAHL-D, SAHL-D22, NVS-D, vocabulary and prose literacy**

	Rec22	Com22	SAHL-D22	Rec	Com	SAHL-D	NVS-D	Voc	Prose
Recognition22	-								
Comprehension22	0.59	-							
SAHL-D22	0.88	0.90	-						
Recognition	0.98	0.58	0.87	-					
Comprehension	0.64	0.96	0.90	0.63	-				
SAHL-D	0.87	0.88	0.98	0.87	0.93	-			
Newest Vital Sign	0.49	0.44	0.52	0.48	0.48	0.53	-		
Vocabulary	0.50	0.54	0.58	0.53	0.57	0.61	0.30	-	
Prose literacy	0.55	0.48	0.57	0.53	0.52	0.58	0.49	0.55	
HLSEU		0.25**	0.21**		0.27**	0.22**	0.20*	0.20*	0.17*

Rec = recognition; Com = comprehension; NVS-D = Newest Vital Sign, Dutch version; Voc = vocabulary; Prose = prose literacy.

All unmarked correlations: p < .001; **p < .01; *p < .05.

n = 272 for all variables except HLSEU (n = 166).

Table 3 shows that the associations between the SAHL-D and prose literacy (model 1), vocabulary (model 2) and NVS-D (model 3) remained significant after correction for differences in educational level, age, gender, and working in health care. The association between SAHL-D and subjective HL disappeared after those adjustments (model 4); the association between SAHL-D and educational level remained significant after adjustment for age, gender and working in health care (model 5).

**Table 3:** **Regressing SAHL-D scores on educational level, demographic and literacy variables (standardized B; 95% CI)**

	Model 1	Model 2	Model 3	Model 4	Model 5
Prose literacy	**1.01** 0.80/1.22				
Vocabulary		**0.71** 0.61/0.82			
Objective HL (NVS-D)			**1.80** 1.36/2.23		
Subjective HL (HLS-EU)				0.09 -0.03/0.21	
Middle education level (ref = low)	**1.74** 0.08/3.74	1.21 -0.30/2.71	**1.20** -0.53/2.92	**3.11** 0.82/5.40	**2.07** 0.15/3.99
High education level (ref = low)	**4.64** 2.82/6.46	**3.53** 1.87/5.20	**4.95** 3.07/6.83	**7.71** 5.42/9.99	**7.48** 5.49/9.47
Age	**0.08** 0.03/0.13	**-0.05** -0.09/-0.01	**0.11** 0.06/0.16	0.00 -0.07/0.06	0.04 -0.01/0.09
Gender	**2.57** 1.19/3.95	**3.65** 2.40/4.90	**1.95** 0.51/3.39	**2.48** 0.49/4.47	**2.88** 1.29/4.47
Worked in health care in (ref = never)	0.74 -1.01/2.49	1.24 -0.34/2.82	1.33 -0.47/3.15	**2.51** 0.14/4.88	1.03 -0.99/3.06
Now works in health care (ref = never)	**4.13** 2.30/5.96	**3.35** 1.69/5.01	**3.76** 1.86/5.65	**4.06** 1.64/6.48	**4.48** 2.37/6.59
Adjusted R^2^ (SE)	0.444 (5.16)	0.548 (4.65)	0.406 (5.33)	0.332 (5.52)	0.257 (5.96)

[Significant differences (p < 0.05) are presented in bold].

We determined the potential of the SAHL-D and SAHL-D22 to correctly identify individuals with adequate and inadequate HL. Inadequate literacy was defined as a prose literacy correct score of 6 or lower. This threshold was chosen to be well below the mean correct score for the lowest educational level (8.3); under this definition, 18% of the participants is inadequately literate.

The area under the ROC curve was 0.80 (CI 0.73-0.88) for SAHL-D. In the various uses of SAHIL, we may choose different cutoffs, i.e. the SAHL-D score below which the test taker is considered to be inadequately health literate. High cut-offs help to correctly identify low literacy (as not many of the low-literacy participants reach the threshold), but are not useful in identifying adequate literacy levels as many literate participants do not reach the threshold either. Reversely, low cut-off points better identify adequately literate individuals, but fare badly in detecting low literacy, as a considerable number of low-literacy participant outscore the threshold. Optimal cutoffs are to be found in the middle of the curve. For example, a cut-off score of 52.5 would correctly classify 66% of the test takers with inadequate HL as such and 86% of the test takers with adequate HL. For a cut-off value of 54.5 these values are 74% and 76% respectively; a cut-off of 55.5 gives values of 80% and 69%. While a high detection rate for low literacy seems preferable, higher cutoffs also imply larger numbers of false positives (i.e. people incorrectly ‘diagnosed’ with low literacy). The final cutoff choice depends on the use of the test, and the priorities in a given setting, especially the estimated costs of false-positive and false-negative results.

## Discussion

Like other objective HL measures, the SAHL-D remains close to the basic literacy concept. The REALM [10] and Medic Achievement Reading Test (MART) [24] check the pronunciation of words. The Test Of Functional Health Literacy in Adults (TOHFLA) [25] uses cloze testing of short text passages and numeracy tasks, and the NVS [21] asks questions related to the comprehension of a nutrition label. All these measures were validated against equally basic measures, often other word recognition and cloze tests. The narrow scope of operational HL measures is not surprising. First, HL measures are often designed in response to the practical demand for tests that can be quickly administered. Second, activities such as accessing, appraising and applying information are harder to test objectively than understanding information, i.e. they are generally examined by means of self-assessment questions. Although Pander Maat & Lentz [26] found a substantial correlation between a health-vocabulary test and success in answering questions about medicine information leaflets, the relation between general and domain-specific literacy is still unclear. As prose (and document) literacy provide the ability to acquire new knowledge where needed, and individuals will often need to process new medical information, a general literacy test seems to be a sensible indication of HL. Nevertheless, from a face validity point of view, it is advisable to use health-related stimuli in literacy tests administered in the health domain. Furthermore, as argued by Baker [27], the distinction between general reading fluency and health-related reading fluency is important for research because a health-related literacy measure is likely to be more closely related to health outcomes than a general literacy measure.

A strength of this study is that the SAHL-D was based on a careful selection and pretest of health-related words that are frequently applied in The Netherlands. Considerable effort was required to find items that were sufficiently demanding for the test, given that Dutch has a fairly transparent orthography; this may explain why the earlier REALM-D test was less successful. Furthermore, adding a comprehension component to the test yielded more discriminative power, at least in the more demanding format used in the main study.

A limitation of the present study is that, in the validation study, the sample was restricted to persons able to write and speak Dutch and having access to internet. This probably means that on average, our research sample is somewhat more literate than the general population. Therefore, we recommend that the SAHL-D be implemented in various clinical contexts and different populations to further investigate its reliability and validity. Another limitation is that there is no objective (health) literacy test available in Dutch. We therefore used an item sample taken from prose literacy tests used in Dutch higher secondary education. Since cut-off points were not available for these items, we defined adequate and inadequate prose literacy with reference to the mean proportion for the lowest educational group.

## Conclusion

The SAHL-D represents a new HL assessment tool in Dutch, consisting of a recognition and comprehension test for 33 (or 22) health-related words. The results of the first validation study provide positive evidence for the reliability and validity of the SAHL-D.

As hypothesized, we found a strong correlation between SAHL-D with general vocabulary, prose literacy and the NVS-D; substantial correlations were found between all literacy measures, ranging from 0.53-0.61. We expected a significant (but not sizeable) correlation between the SAHL-D and the HLS-EU-Q16, since HL is subjectively measured in the HLS-EU and the SAHL-D is an objective measure; in fact a lower correlation was found between the SAHL-D and the HLS-EU-Q16, that was not significant after correction for educational level and other background variables. As expected we found a significant correlation between the SAHL-D and educational level and age; the correlation with education being stronger than that with age. All these results support the construct validity of the SAHL-D. After adjustment for educational level, age was no longer significant in the regression model, indicating that differences in age could be explained by differences in educational level.

Although we did not expect gender differences in SAHL-D scores, our regression analyses found that women scored higher than men, also after correcting for age and educational level. As our general vocabulary and prose literacy scores show no gender differences, this difference seems to be specific to the health domain. Discussion of related evidence can be found in Peerson & Saunders [28].

In conclusion, our results indicate that the SAHL-D is a valid Dutch-language measure of functional HL that can be applied in research on the role of objective HL in health and healthcare use, the differential effectiveness of (preventive) health intervention by HL, and the development of targeted interventions in healthcare. Implementation of the SAHL-D in various contexts in public health and health care is necessary to further investigate its reliability and validity.

## Electronic supplementary material

Additional file 1: **Words included in the SAHL-D.** (PDF 80 KB)

Additional file 2: **Sample items of the SAHL-D comprehension test.** (PDF 83 KB)

## References

[1] Stahl S, Hiebert EH, Paris SG, Stahl SA (2005). A problem for reading comprehension assessment. Children’s reading comprehension and assessment.

[2] White S (2011). Adult functional literacy. Connecting text features, task demands and participant skills.

[3] Remund D (2008). Financial Literacy Explicated: The Case for a Clearer Definition in an Increasingly Complex Economy. J Consum Aff.

[4] Hobbs R, Frost R (2003). Measuring the acquisition of media-literacy skills. Read Res Q.

[5] Ratzan SC, Parker RM, Selden CR, Zorn M, Ratzan SC, Parker RM (2000). Introduction. National Library of Medicine Current Bibliographies in Medicine: Health Literacy.

[6] Mancuso JM (2009). Assessment and measurement of health literacy: an integrative review of the literature. Nurs Health Sci.

[7] Jordan JE, Osborne RH, Buchbinder R (2011). Critical appraisal of health literacy indices revealed variable underlying constructs, narrow content and psychometric weaknesses. J Clin Epidemiol.

[8] Sørensen K, Van den Broucke S, Fullam J, Doyle G, Pelikan J, Slonska Z, Brand H (2012). Health literacy and public health: A systematic review and integration of definitions and models. BMC Public Health.

[9] Nutbeam D (2000). Health literacy as a public health goal: a challenge for contemporary health education and communication strategies into the 21th century. Health Promot Int.

[10] Davis TC, Long SW, Jackson RH, Mayeaux EJ, George RB, Murphy PWY, Crouch MA (1993). Rapid estimate of adult literacy in medicine: a shortened screening instrument. Fam Med.

[11] Fransen MP, Van Schaik TM, Twickler TB, Essink-Bot ML (2011). Applicability of internationally available health literacy measures in The Netherlands. J Health Commun.

[12] Nurss JR, Baker DW, Davis TC, Parker RM, Williams M (1995). Difficulties in functional health literacy screening in Spanish-speaking adults. Journal of Reading.

[13] Lee SY, Bender DE, Ruiz RE, Cho YI (2006). Development of an easy-to use Spanish health literacy test. Health Serv Res.

[14] Lee SY, Stucky BD, Lee JY, Rozier RG, Bender DE (2010). Short assessment of health literacy – Spanish and English: a comparable test of health literacy for Spanish and English speakers. Health Serv Res.

[15] *Thesaurus zorg en welzijn*. [http://www.thesauruszorgenwelzijn.nl]

[16] Stichting Cito Instituut voor Toetsontwikkeling (2005). Exam 8 higher secondary education. [Adviestoets havo/vwo, toets 8].

[17] Keselman A, Tse T, Cromwell J, Browne A, Ngo L, Zeng Q (2007). Assessing consumer health vocabulary familiarity: an exploratory study. J Med Internet Res.

[18] Brabers A, Reitsma-van Rooijen M, De Jong D (2012). Consumers panel health care: Basic report with information on the panel [Consumentenpanel Gezondheidszorg: Basisrapport met informatie over het panel].

[19] UNESCO Institute for Statistics (2014). ISCED: International standard classification of education.

[20] HLS-EU Consortium: *Comparative Report on Health Literacy in Eight EU Member States*. [http://www.health-literacy.eu]

[21] Weiss BD, Mays MZ, Martz W, Castro KM, DeWalt DA, Pignone MP (2005). Quick assessment of literacy in primary care: the newest vital sign. Ann Fam Med.

[22] OECD, Statistics Canada (2011). Literacy for Life: Further Results from the Adult Literacy and Life Skills Survey.

[23] Houtkoop W, Allen J, Buisman M, Fouarge D, Van der Velden R: *Kernvaardigheden in Nederland: Resultaten van de Adult Literacy and Life Skills Survey (ALL) (Basic abilities in The Netherlands: Results of the Adult Literacy and Life Skills Survey (ALL))*. http://www.piaac.nl/_images/user/20131008093428ecbo.12-123_Kernvaardigheden_in_NederlandA00686.pdf (accessed August 8, 2014)

[24] Hanson-Divers EC (1997). Developing a medical achievement reading test to evaluate patient literacy skills: a preliminary study. J Health Care Poor Underserved.

[25] Parker RM, Baker DW, Williams MV, Nurss JR (1995). The test of functional health literacy in adults: a new instrument for measuring patients’literacy skills. J Gen Intern Med.

[26] Pander Maat HLW, Lentz L (2010). Improving the usability of patient information leaflets. Patient Educ Couns.

[27] Baker DW (2006). The meaning and the measure of health literacy. J Gen Intern Med.

[28] Peerson A, Saunders M (2009). Men’s health literacy: Advancing evidence and priorities. Crit Pub Health.

[CR29] The pre-publication history for this paper can be accessed here:http://www.biomedcentral.com/1471-2458/14/990/prepub

