# Supplementary material for: A short assessment of health literacy (SAHL) in the Netherlands
Source: BMC Public Health. 2014 Sep 23;14:990. doi: 10.1186/1471-2458-14-990 (PMC4190424; doi:10.1186/1471-2458-14-990)
Supplement: Supplementary file 1 — Additional file 1: Words included in the SAHL-D. (PDF 80 KB) [file 12889_2014_7114_MOESM1_ESM.pdf]

Additional file 1. Words included in the SAHL-D

*Words left out in the SAHL-D22

	Dutch	English translation	Topic area
1.	Achillespees*	Achilles' tendon	Body part or bodily function
2.	Adrenaline	Adrenaline	Body part or bodily function
3.	Apathie	Apathy	Diseases
4.	Apneu*	Apnea	Diseases
5.	Bètablokker	Beta blocker	Tests and treatments
6.	Biopsie	Biopsy	Tests and treatments
7.	Chiropractor	Chiropractor	Specializations
8.	Chlamydia	Chlamydia	Diseases
9.	Defibrillatie	Defibrillation	Tests and treatments
10.	Delirium	Delirium	Diseases
11.	Dwarslaesie*	Paraplegia	Diseases
12.	Echografie*	Echography	Tests and treatments
13.	Euforie	Euphoria	Diseases
14.	Geleermiddel	Gelling agent	Tests and treatments
15.	Hemofilie	Hemophilia	Diseases
16.	Hospice*	Hospice	Tests and treatments
17.	Malaise*	Malaise	Diseases
18.	Manisch*	Manic	Diseases
19.	Obesitas	Obesity	Diseases
20.	Oedeem*	Oedema	Diseases
21.	Oncologie*	Oncology	Specializations
22.	Orthodontie	Orthodontics	Specializations
23.	Palliatief	Palliative	Tests and treatments
24.	Pancreas	Pancreas	Body part or bodily function
25.	Pessarium	Pessary	Tests and treatments
26.	Plaque	(Dental) plaque	Diseases
27.	Prenataal*	Prenatal	Tests and treatments
28.	Psoriasis	Psoriasis	Diseases
29.	Reflux	Reflux	Diseases
30.	Resistent*	Resistant	Tests and treatments
31.	Schilfering	Flaking	Diseases
32.	Schizofrenie	Schizophrenia	Diseases
33.	Ventrikel	Ventricle	Body part or bodily function
