# Supplementary material for: A short assessment of health literacy (SAHL) in the Netherlands
Source: BMC Public Health. 2014 Sep 23;14:990. doi: 10.1186/1471-2458-14-990 (PMC4190424; doi:10.1186/1471-2458-14-990)
Supplement: Supplementary file 2 — Additional file 2: Sample items of the SAHL-D comprehension test. (PDF 83 KB) [file 12889_2014_7114_MOESM2_ESM.pdf]

Additional file 2 Sample items of the SAHL-D comprehension test (translated from Dutch)
Correct alternatives are in bold font; the 'related' distractor is underlined.

Orthodontics

- **a. Specialty concerned with irregularities in the teeth**
- b. Specialty concerned with the prevention of cavities in teeth
- c. Specialty concerned with jaw surgery
- d. I do not know

Psoriasis

- **a. Disease leading to red dry spots on the skin**
- b. Brown spots, especially on the face
- c. Dry skin because of a lack of sebaceous glands
- d. I do not know
